# Therapeutic effect of hepatocyte growth factor-overexpressing bone marrow-derived mesenchymal stem cells on CCl_4_-induced hepatocirrhosis.

**DOI:** 10.1038/s41419-018-1239-9

**Published:** 2018-12-11

**Authors:** Yichi Zhang, Ruini Li, Weiwei Rong, Mingzi Han, Chenghu Cui, Zhenning Feng, Xiaoli Sun, Shizhu Jin

**Affiliations:** Department of Gastroenterology and Hepatology, The Second Affiliated Hospital, Harbin Medical University, Harbin, Heilongjiang China

## Abstract

Hepatocirrhosis is one of the most severe complications of chronic hepatic disease in terms of medical intervention, and the available therapies are limited and not very successful. In this study, bone marrow-derived mesenchymal stem cells (BM-MSCs) from host rats were transduced with an adenoviral vector labelled with green fluorescent protein (EGFP) to overexpress hepatocyte growth factor (HGF). The therapeutic effect of these modified stem cells (HGF-BM-MSC group) transplanted intravenously into hepatocirrhosis model rats treated with CCl_4_ was evaluated using serological, biochemical and histological approaches. We compared the rats in the HGF-BM-MSC group with those in the other groups (rats treated with BM-MSCs, rats treated with HGF and untreated rats (Controls)) in detail. The localisation of EGFP-tagged BM-MSCs in the injured liver was evaluated using a microscope, and the cells co-expressed hepatocyte nuclear factor 4α, albumin and cytokeratin 18. After treatment for 4 weeks, the HGF-BM-MSC, BM-MSC and HGF groups exhibited increased protein and mRNA levels of hepatocyte nuclear factor 4α, albumin and cytokeratin 18, but decreased levels of aspartate aminotransferase, alanine aminotransferase and total bilirubin. These findings indicate that BM-MSC transplantation and HGF application have great potential for the treatment of hepatocirrhosis.

## Introduction

Cirrhosis is a common hepatic disease that can be caused by chemical injury or viral infection in the liver. Pathologically, this disease is characterised by chronic and progressive hepatocyte degeneration and excessive fibrosis, which may lead to severe clinical outcomes, such as ascites, variceal haemorrhage and encephalopathy^1–3^. Currently, the most effective treatment for hepatic cirrhosis is liver transplantation, but its clinical application is limited due to the shortage of donor material, the requirement of expert technical support, and high-hospital costs.

Stem cell therapy may offer a new hope for the treatment of many chronic degenerative diseases. Bone marrow-derived mesenchymal stem cells (BM-MSCs) are deemed ideal for transplantation, because they can differentiate into different cell types, including hepatocytes, myocardial cells and neurons^4–6^. Many studies have shown that BM-MSCs differentiate into hepatic cells in external and internal conditions^7,8^, whereas other reports have demonstrated the potential therapeutic utility of BM-MSCs in the treatment of chronic liver diseases^9–11^.

Certain cell growth factors have value in the treatment of diseases in clinical practice. Specifically, hepatocyte growth factor (HGF), originally discovered from patients with hepatitis, has been shown to be widely expressed in liver, spleen, kidney, lung, heart and other organs^12^. HGF has a trophic effect on many types of cells, such as epithelial, endothelial and stromal cells, by promoting mitotic activity, growth and migration and preventing apoptosis^13,14^. In the liver, HGF can stimulate hepatocyte proliferation, inhibit hepatocyte apoptosis, and thus promote liver regeneration following injury^15^.

Previously, we demonstrated that BM-MSC transplantation may promote the repair process of the liver following chemical injury in rats^16,17^. HGF could be given as an adjunctive agent during stem cell therapy to enhance the effect of tissue regeneration, but the use of an extrinsic source of HGF is costly, particularly when it needs to be replenished constantly. Genetically engineering HGF into BM-MSCs to treat liver cirrhosis would be optimal from both pharmacological and practical perspectives. Therefore, the aim of this study was to determine whether BM-MSCs that overexpress HGF provide better therapeutic effects than regular BM-MSCs on hepatocirrhosis induced by carbon tetrachloride (CCl_4_) in rats. We examined the localisation of the transplanted BM-MSCs with enhanced HGF expression in liver tissues and compared their therapeutic efficacy with that found in four other experimental groups to determine whether HGF-modified BM-MSCs could improve liver cirrhosis in rats.

## Results

### CCl_4_-induced cirrhotic pathology in rat liver

**Fig. 1:**
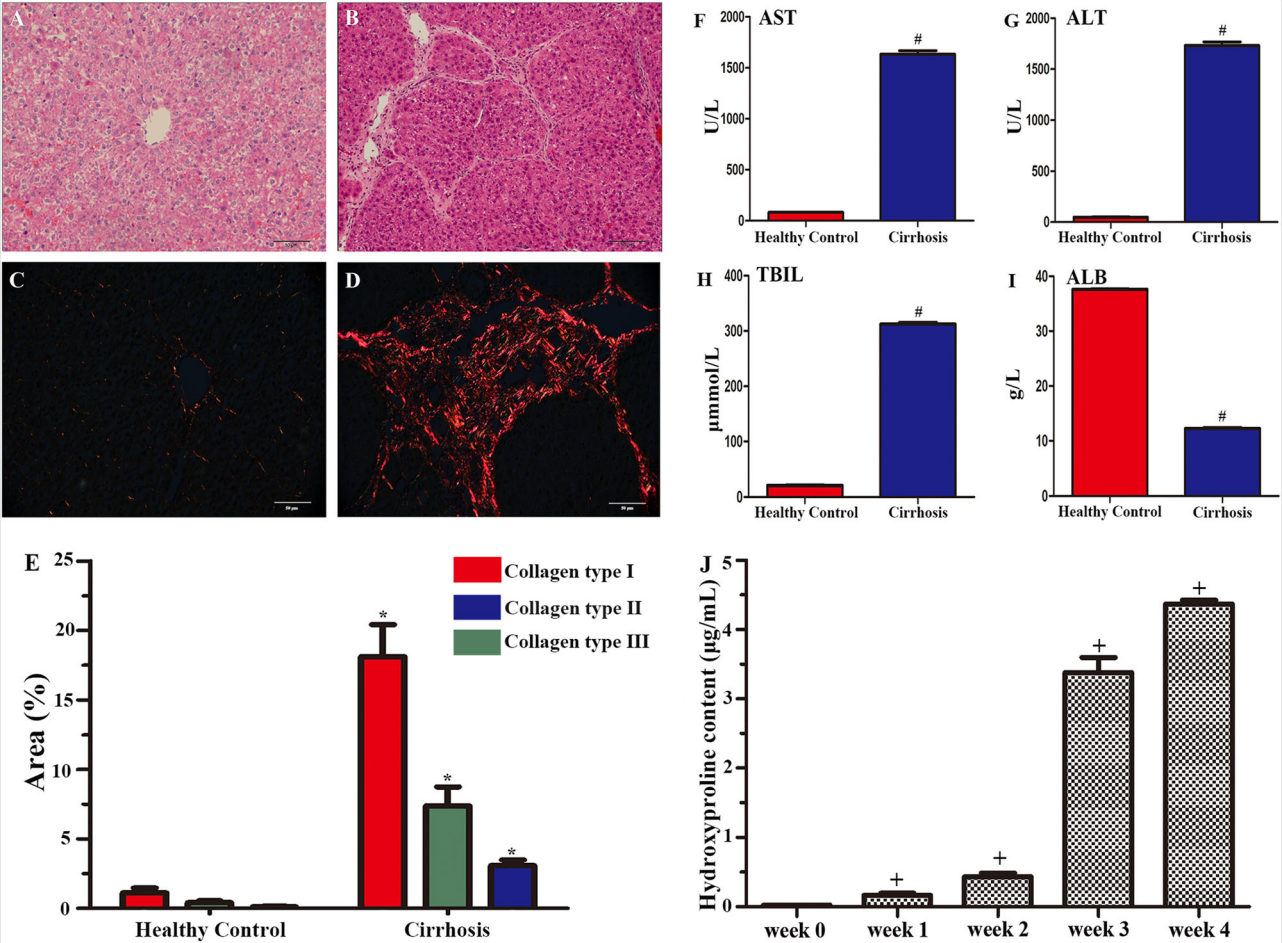
Histological analysis, serological characterisation and hydroxyproline determination of carbon tetrachloride (CCl_4_)-induced liver injury. **a** and **b** show H&E staining of liver tissues from a healthy control **a** and a CCl_4_-treated rat **b** 4 weeks after model induction. Picrosirius red staining in liver sections from a healthy control and a rat with cirrhosis 4 weeks after model induction **c**, **d**. Quantitative analysis of fibrillary collagen showed significant increases, with the highest increase in collagen type I, compared with that in healthy controls **e**. Scale bar = 50 μm in **a** applied to **b**–**d**. All the data are displayed as the means ± SDs; *n* = 4 for each group, *p < 0.05 vs. healthy controls. **f**–**i** show dramatic increases in serum alanine aminotransferase (AST) **f**, aspartate aminotransferase (ALT) **g** and total bilirubin (TBIL) **h** in the CCl_4_-treated rats. In contrast, serum levels of albumin (ALB) were lower in the CCl_4_-treated group than the control group **i**. All the data are displayed as the means ± SDs; *n* = 5 for each group, #*p* < 0.001 vs. healthy controls. **j** shows dramatic increases in serum hydroxyproline content in 4 weeks. All the data are displayed as the means ± SDs the data; *n* = 5 for each group, +*p* < 0.001 vs. week 0

We verified the establishment of the animal model of cirrhosis by observations of haematoxylin and eosin (HE)-stained liver sections from rats that survived 1 week following CCl_4_ or saline (healthy control) treatments. Compared to healthy controls (Fig. 1a), the experimental animals showed cell degeneration, necrosis, fibrous proliferation and false flocculus in the liver sections (Fig. 1b). The deposition of fibrillary collagen was visualised using picrosirius red staining in healthy controls (Fig. 1c) and the cirrhosis group (Fig. 1d), and the fibrillary collagen content was determined by serum hydroxyproline content analysis (Fig. 1j). Fibrillary collagen (types I and III) was clearly observed in tissues without the need for exogenous labelling. Quantitative analysis indicated that fibrillary collagen was drastically increased (*p* < 0.05), with the highest increase in collagen type I, compared to that of the healthy controls (Fig. 1e). Hydroxyproline is an amino acid that is stably deposited in the liver and is only associated with collagenous connective tissue; thus, it is a good substitute for the quantitative determination of collagen deposition^18^. Compared with healthy controls, the cirrhosis group showed significant increases in hydroxyproline content (*p* < 0.001) in the fourth week (Fig. 1j). Serological tests showed significant elevation of aspartate aminotransferase (AST) (Fig. 1f), alanine aminotransferase (ALT) (Fig. 1g) and total bilirubin (TBIL) (Fig. 1h) in blood from the experimental group relative to that from healthy controls (all *p* < 0.01). In contrast, serum albumin (ALB) was lower in the experimental group than the healthy controls (Fig. 1i).

### Virus-incorporated BM-MSCs exhibited bone marrow stem cell antigenic markers

**Fig. 2:**
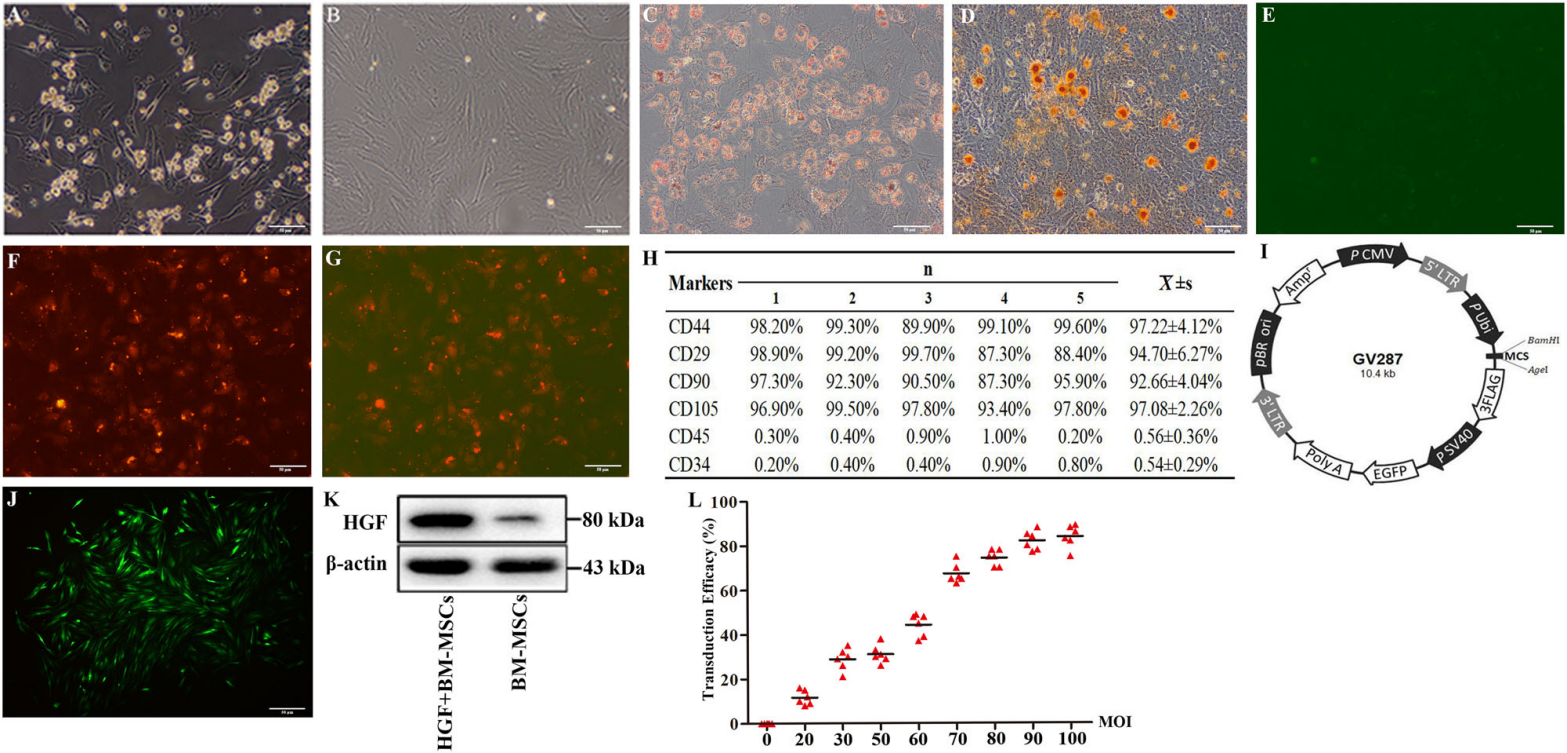
Morphology and antigenic phenotyping of the BM-MSCs modified with the rat hepatic growth factor gene (rHGF). The BM-MSCs were small and spherical in the first 24 h of culture **a**. The cells increased in number and size in the following 2–3 days of culture **b**. Osteogenic and adipogenic differentiations are shown in **c** and **d**. Negative results of Dil-Ac-LDL uptake and bound FITC-CEA-1 are shown in **e**–**g**. **h** summarises the flow cytometric results of BM-MSC-specific antigenic markers. **i** shows the construction of the adenoviral vector that contains the hepatic growth factor gene (HGF) and green fluorescent protein reporter gene (EGFP). **j** shows the transduced BM-MSCs at the third passage. The cells exhibit a spindle/fusiform shape and express bright green fluorescence. A western blot analysis of EGFP-positive cells overexpressing HGF is shown in **k**. The dynamic changes between transduction efficiency and multiplicity of infection (MOI) are shown in **l**. Scale bar = 50 μm in **a**–**g** applied to **j**

Cultured BM-MSCs exhibited morphological irregularities at 3–4 days of in vitro expansion (Fig. 2a) and were largely spindle-like by the third passage (Fig. 2b). Osteogenic and adipogenic differentiation induction was performed to assess the multipotency of the cultured BM- MSCs. After 4 weeks of culture in osteogenic and adipogenic differentiation media, the cells differentiated into osteogenic and adipogenic lineages, as demonstrated through Alizarin red (Fig. 2c) and Oil Red O staining (Fig. 2d). Negative results for Dil-Ac-LDL uptake and FITC-CEA-1 binding affinity indicated that these cells were not endothelial progenitor cells (Fig. 2e–g). To assess the maintenance of bone marrow cell antigenic phenotypes, we carried out fluorescence-activated cell sorting (FACS) analysis. The FACS results indicated that 97.22% of the cells expressed CD44, 94.7% expressed CD29, 92.66% expressed CD90, and more than 97.08% of the cells expressed CD105, all of which are surface markers of bone marrow stem cells. In contrast, less than 0.56% of cultured cells expressed CD45, whereas 0.54% expressed CD34, the surface antigens of leucocytes and haematopoietic cells, respectively (Fig. 2h). The *EGFP* gene constructs in the viral vector were used to assess the transduction efficiency of the HGF-expressing virus in cultured BM-MSCs (Fig. 2i). The majority of BM-MSCs at the third passage exhibited bright green fluorescence (Fig. 2j). The transduction efficiency was evaluated by the ratio of EGFP-positive cells to the total cell number (Fig. 2l). Overall, most cells transduced with the viral vector remained BM-MSCs at the third passage, and EGFP^+^ cells exhibited HGF overexpression (Fig. 2k)

### EGFP-expressing BM-MSCs were present in injured livers following transplant

**Fig. 3:**
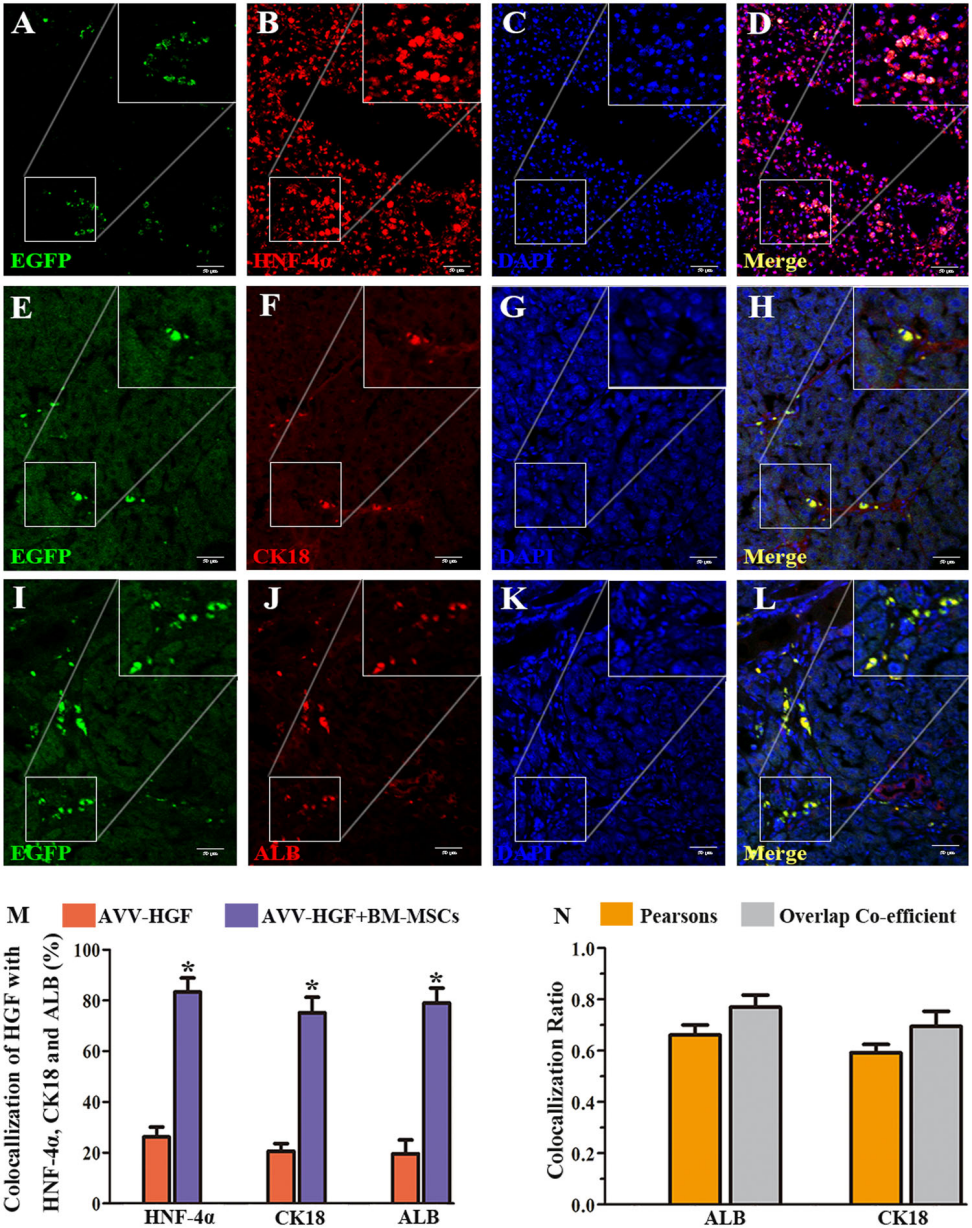
Co-localisation of rHGF-modified BM-MSCs with hepatocytic markers in liver sections from a CCl_4_-treated rat. The cell showing green fluorescence represents BM-MSCs that were transduced with the viral vector carrying the rHGF gene (left panels). These cells were immunofluorescently co-labelled for HNF-4α **a**–**d**, CK18 **e**–**h** and ALB **i**–**l** with Alexa Fluor® 594-conjugated donkey secondary antibodies. Scale bar = 50 μm in **a** and is applied to other panels. A statistical comparison of the percentages of double-stained cells in HGF-positive cells between the HGF and HGF-BM-MSC groups is shown in **m**. All the data are displayed as the means ± SDs; *n* = 5 for each group, *p < 0.001 versus the AVV-HGF group. The graph format (N) shows Pearson’s correlation and the overlap coefficient of liver sections co-stained with EGFP, ALB and CK18. All the data are displayed as the means ± SDs; *n* = 5 for each group

To confirm the presence of EGFP-expressing BM-MSCs in the injured liver following transplantation, we performed immunofluorescence labelling for hepatocyte nuclear factor 4α (HNF-4α), cytokeratin (CK18) and ALB in liver sections from animals with CCl_4_ lesions that survived 2 (not shown) and 4 weeks (Fig. 3) after bone marrow cell transplantation. EGFP fluorescent cells (green fluorescent) were clearly present in the liver sections. Importantly, these cells were mostly co-labelled for HNF-4α, indicating the strong expression of this hepatic transcription factor (red fluorescence, Fig. 3a–d). In addition, these EGFP fluorescent cells were found to predominantly express the hepatic cell markers CK18 (Fig. 3e–h) and ALB (Fig. 3i–l), indicating the potential development of the stem cells into hepatic cells. Statistical comparisons of the percentages of HNF-4α, CK18 and ALB double-stained cells in the HGF-positive cells between the HGF and HGF-BM-MSC groups were performed (Fig. 3m). The percentage of double-stained cells in the HGF-positive cells of the HGF-BM-MSC group was significantly higher than that in the HGF group (all *p* < 0.001). Pearson’s correlation and the overlap coefficient of the liver sections co-stained for EGFP, ALB and CK18 are shown (Fig. 3n).

### Phenotypic characterisation of the HGF gene-modified BM-MSCs that differentiated into hepatocyte-like cells

**Fig. 4:**
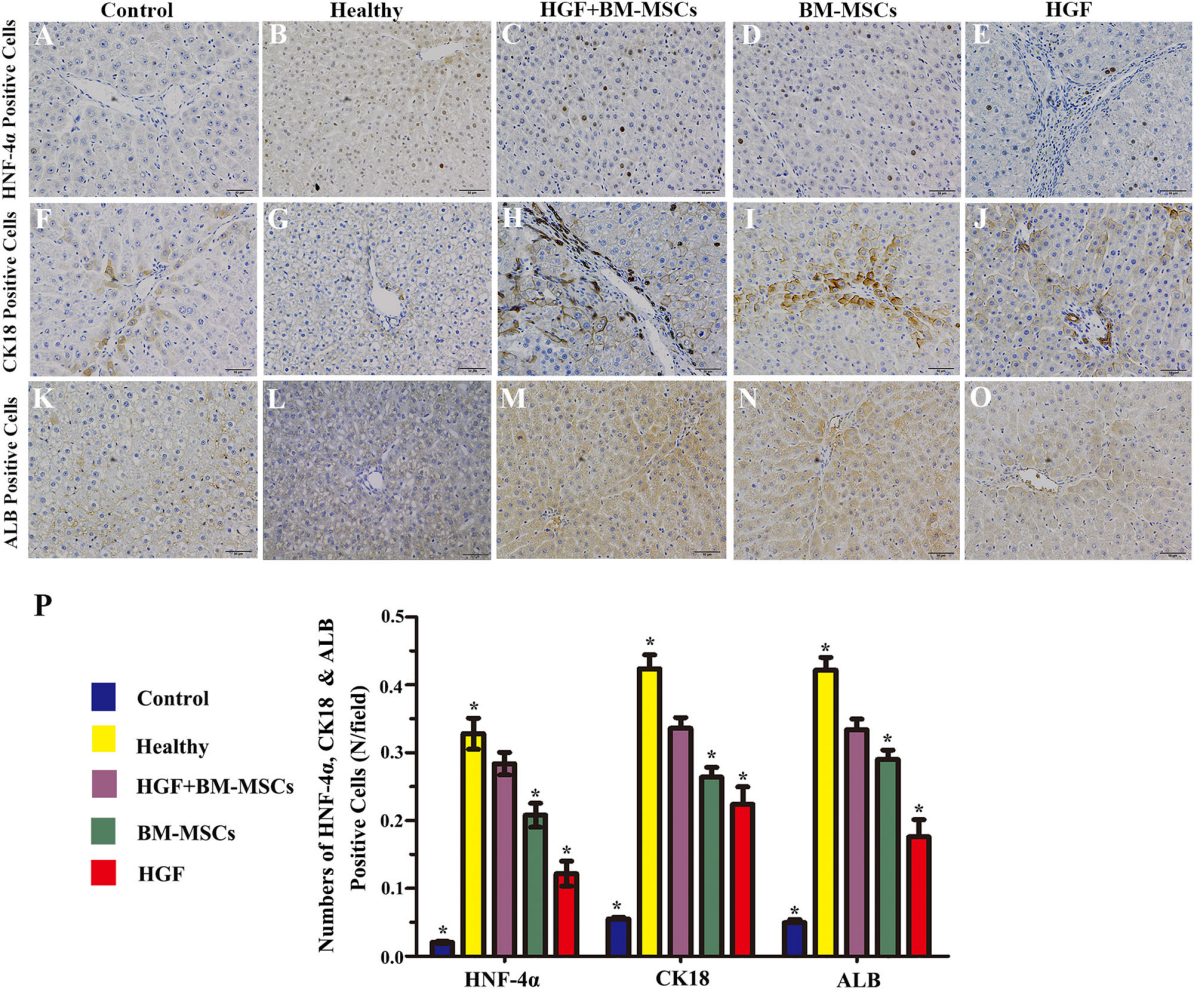
Phenotypic characterisation of rHGF gene-modified BM-MSCs that differentiated into hepatocyte-like cells. The dramatic increases of HNF-4α- **a**–**e**, CK18- **f**–**j** and ALB-positive cells **k**–**o** are shown in the panels. Quantitative analysis indicated that the levels of the HGF-BM-MSC group were significantly higher than those of the control, BM-MSC and HGF groups (all *p < 0.05) **p**. Scale bar = 50 μm in **a** applied to the other panels

Among the 4 treatment groups, immunohistochemical analysis showed that HNF-4α-, CK18- and ALB-positive cells were most common in the liver tissues from rats in the HGF-BM-MSC group (Fig. 4a–o). Quantitative analysis showed that the control group had the fewest HNF-4α-, CK18- and ALB-positive cells, whereas the healthy group had the highest numbers of these cells. The values for the HGF-BM-MSC group were significantly higher than those of the other two groups (all *p* < 0.05) (Fig. 4p).

### HGF and bone marrow stem cell therapy promoted hepatic protein expression

**Fig. 5:**
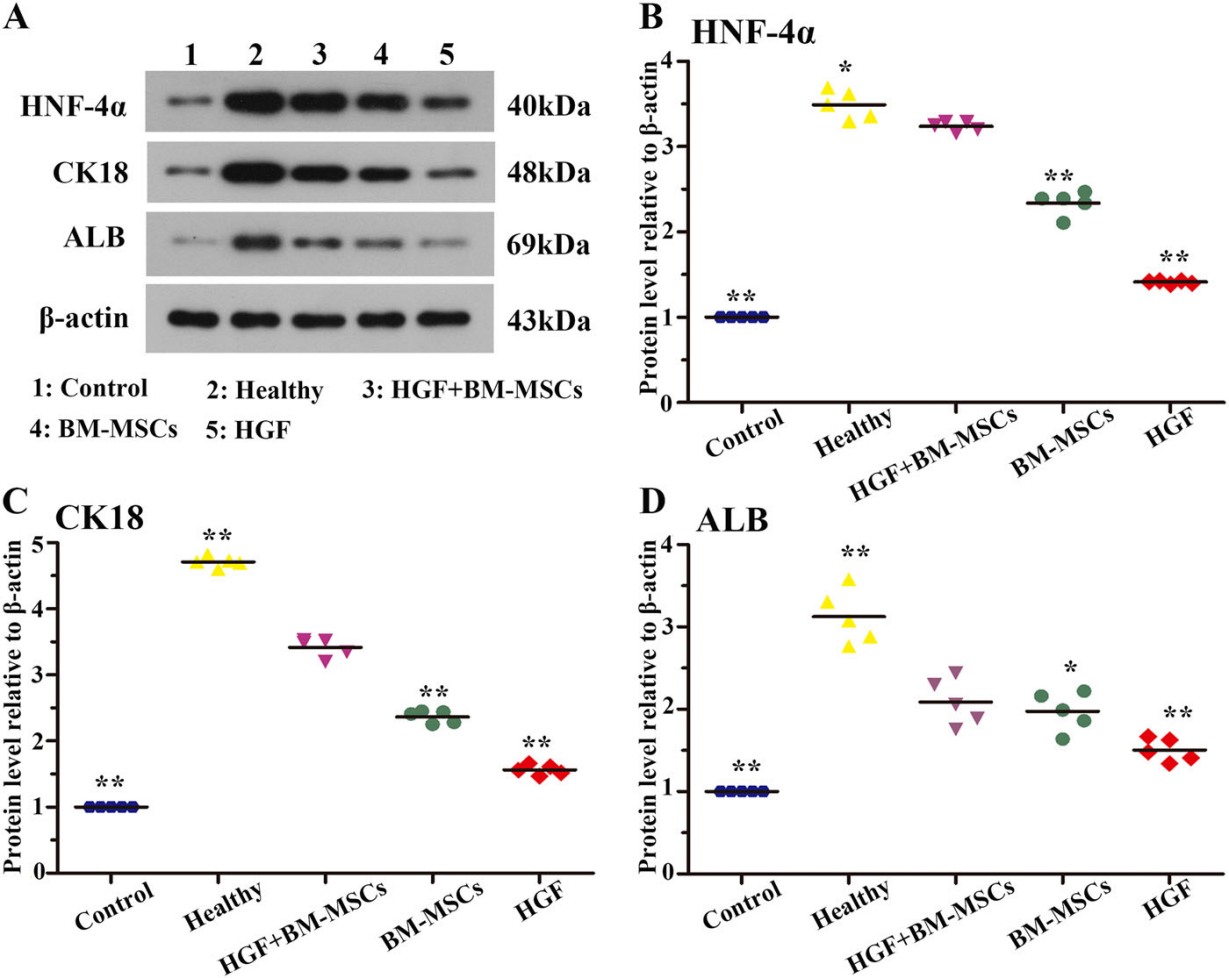
Effects of rHGF gene-modified BM-MSCs on hepatic protein expression. **a** shows a representative western blot image of HNF-4α, CK18 and ALB relative to the internal standard β-actin. Quantities are shown in the **b**–**d** for the above markers expressed as ratio to the control groups. Levels of the hepatic proteins were highest in the HGF-BM-MSC-treated group relative to the HGF, BM-MSC and saline-treated groups (all *p < 0.05, **p < 0.001)

We compared the effects of BM-MSCs with enhanced HGF expression to those of the BM-MSC and HGF treatment groups following liver injury. For this analysis, we performed a biochemical analysis of the hepatic cell proteins HNF-4α, CK18 and ALB in tissue lysates. The levels of these proteins were determined by immunoblot analysis of animals that survived 4 weeks after the intravenous treatments. The HNF-4α protein levels were elevated in liver lysates of the healthy, HGF, BM-MSC and HGF-BM-MSC groups relative to the controls, with the highest level observed in the healthy group (Fig. 5a). Quantitative analysis revealed a significant difference in the means of the groups (*p* < 0.05), with a post hoc test showing significant differences between the HGF-BM-MSC group and all other groups (Fig. 5b). Similarly, the immunoblot signal for CK18 was dramatically increased in the HGF-BM-MSC group relative to the HGF, BM-MSC and control groups (Fig. 5c). Densitometry showed significant differences in CK18 protein levels in the HGF-BM-MSCs group relative to the remaining groups (Fig. 5c) (*p* < 0.001). Moreover, ALB levels in liver lysates were highest in CCl_4_-injured rats treated with HGF-BM-MSC, and the levels in this group were significantly different from the levels in the HGF, BM-MSC and control groups (Fig. 5d) (*p* < 0.05).

### HGF and bone marrow stem cell therapy promoted hepatic mRNA expression

**Fig. 6:**
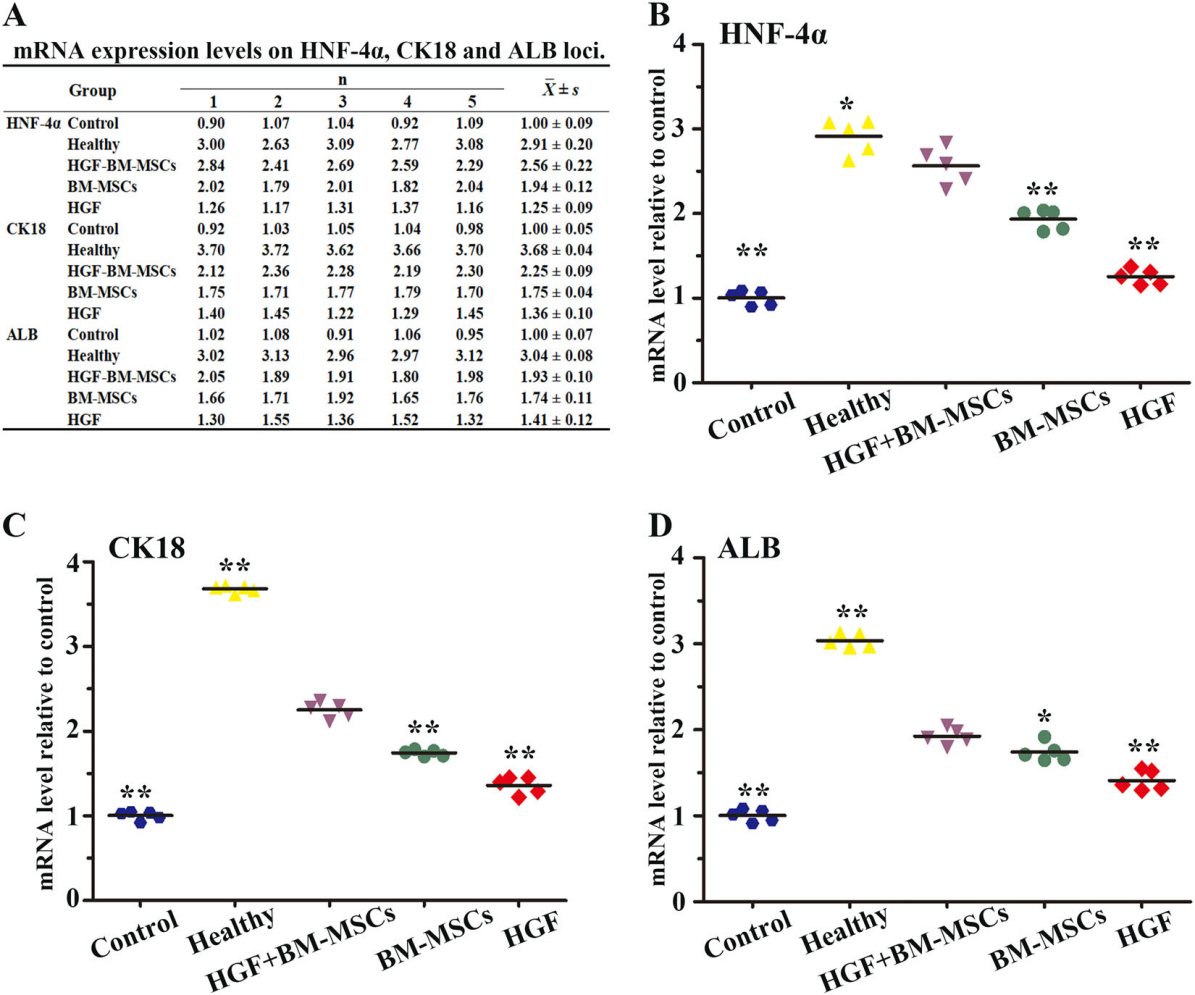
Effects of rHGF gene-modified BM-MSCs on the mRNA expression of selected hepatic proteins. The quantitative data of the HNF-4α, CK18 and ALB mRNA expression in different groups are shown **a**. All the data are displayed as the means ± SDs. Quantitative analyses of the above markers are illustrated in **b**–**d**, with the data expressed as a ratio of β-actin expression. The mRNA expression was highest in the HGF-BM-MSC-treated group relative to the HGF, BM-MSC and control groups (all *p < 0.05, **p < 0.01)

The mRNA levels of the above hepatic proteins were also assayed in liver tissues from the 5 animal groups 4 weeks post therapy using RT-PCR. HNF-4α mRNA expression levels were dramatically increased in the HGF-BM-MSC group compared with those in the BM-MSC, HGF and control groups (Fig. 6b) (all *p* < 0.001). Statistical analysis indicated that the HGF-BM-MSC group expressed the highest levels of CK18 and ALB, with expression values that were significantly different from those of the HGF-treated, BM-MSC-treated and control groups (Fig. 6c, d) (ANOVA; 6C, all *p* < 0.01; 6D, all *p* < 0.05). The data are expressed as the means ± standard deviations (SDs) in Fig. 6a.

### Serological analysis of liver enzymes

**Fig. 7:**
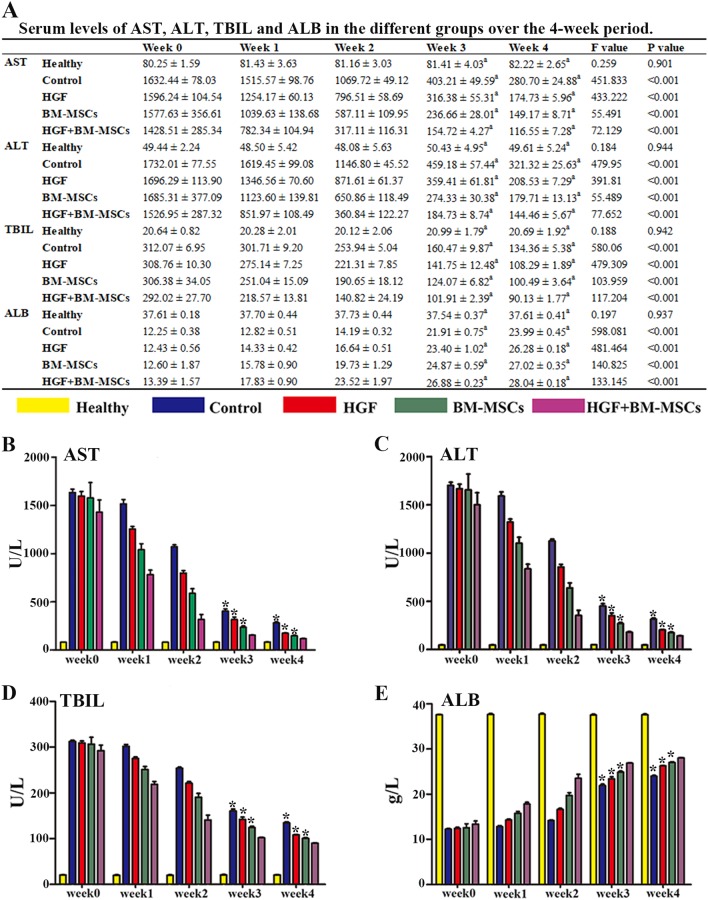
Effects of rHGF gene-modified BM-MSCs on hepatic serological indices monitored during the experimental period (weeks 0–4). Serum levels of aspartate aminotransferase (AST) **b**, alanine aminotransferase (ALT) **c** and total bilirubin (TBIL) **d** in four treatment groups were high at week 0 and declined at 1–4 weeks post lesion. The results of the serum AST, ALT, TBIL and ALB levels in different groups over time are shown **a**. All the data are displayed as the means ± SDs. ^a^ indicates that the *p*-value is lower than the Bonferroni-corrected threshold of 0.005. The AST, ALT, and TBIL levels were lowest in the rats that received rHGF gene-modified BM-MSCs (HGF-BM-MSC group) at the third and fourth weeks, whereas the ALB level was the highest in this group. These levels were significantly different (all *p < 0.005) from those of the other three treatment groups, as determined by post hoc tests

Plasma AST, ALT, TBIL and ALB were monitored at weekly intervals (0, 7, 14, 21 and 28 days) in the five animal groups using venous blood during the experiment and trunk blood at the end of the 28 days of CCl_4_ injection. Overall, serum AST, ALT and TBIL tended to decrease with time in the four treatment groups during the 1-month experimental time window. The reductions in serum AST, ALT and TBIL were most prominent in the HGF-BM-MSC group among the 4 treatment groups. Specifically, the AST, ALT and TBIL levels in the HGF-BM-MSC group were significantly lower than those of the other 3 groups at the end of the 3rd and 4th weeks, as shown by Bonferroni-corrected tests (Fig. 7b–d). In contrast, serum ALB levels in the HGF-BM-MSC group were significantly higher than in all other treatment groups (Fig. 7e). The results of the statistical analysis are shown in Fig. 7a.

## Discussion

In the present study, liver cirrhosis was induced by intraperitoneal injection of CCl_4_^16,17^. Histological analysis showed cirrhotic pathology, while the serological data were consistent with a progressive change characterised by an initial hepatic functional decline followed by recovery. As shown by the significant increase in fibrillary type I and hydroxyproline content, the hepatocirrhosis model was constructed successfully. To determine whether BM-MSCs with enhanced HGF expression mitigated CCl_4_-induced liver injury and/or promoted liver recovery, we compared the experimental effects in 4 groups of CCl_4_-treated animals using biochemical, histological and serological approaches. The rat-derived BM-MSCs used for transplant therapy were confirmed by analysis of their characteristic expression of mesenchymal stem cell markers (CD29, CD90, CD105 and CD44). Virus incorporation in transplanted BM-MSCs was verified using liver sections from the experimental rats.

The mechanism governing cirrhotic pathogenesis of the liver is still unclear, while the activation of hepatic satellite cells and collagen deposition are believed to be involved in the process of liver fibrosis^19^. The imbalance of extracellular matrix synthesis and degradation is also believed to contribute to liver fibrosis^20^. BM-MSCs can secrete a variety of growth factors and cytokines, including HGF, transforming growth factor-β (TGF-β) and interleukins (ILs)^21^. Early studies also proposed that transplanted BM-MSCs may undergo cellular transdifferentiation and thus develop into hepatic cells for liver regeneration, while paracrine effects have recently been identified as a major benefit of bone marrow stem cell therapy^22^. Thus, by providing soluble tissue/cell growth factors, autologous BM-MSC transplantation can promote liver regeneration and attenuate aberrant fibrosis in the treatment of chronic hepatic diseases, such as cirrhosis^23,24^. The findings in the present study are consistent with the above hypothesis that liver growth factors, especially HGF provided by transplanted bone marrow cells, can significantly promote liver regeneration and functional recovery after injury. Thus, genetically modified BM-MSCs overexpressing rat-HGF (rHGF) promoted the endogenous expression of HNF-4α, CK18 and ALB at both the mRNA and protein levels in the liver. Notably, these cells also promoted functional recovery, as indicated by a more pronounced decline in the levels of the liver injury markers AST, ALT and TBIL compared with the other tested groups.

In the present study, we specifically engineered rHGF into transplanted BM-MSCs based on an understanding of its biological activities. HGF, an important hepatic growth factor, can stimulate hepatocyte proliferation, inhibit hepatocyte apoptosis and promote liver regeneration via its receptor activation^25,26^. Previous studies showed that application of exogenous HGF can reduce apoptosis and fibrosis in the liver in a rat model of cirrhosis^27^. This growth factor may reduce the activation of hepatic satellite cells and collagen deposition in the liver and inhibit TGF-β1 secretion, therefore preventing the progress of liver fibrosis^28^. Another study showed that HGF transfection via vitamin A-coupled liposomes may target hepatic satellite cells; increased expression of HGF in these cells reduced the degree of liver fibrosis^29^. However, exogenous HGF is unstable due to its short half-life in the blood circulation^30^. Maintaining high levels of exogenous HGF requires repeated long-term dosing, which is costly and unaffordable for many patients, especially those in low-income countries.

## Conclusions

This study showed that rat-derived BM-MSC transplantation and rat-HGF administration attenuated liver injury or promoted liver recovery in a rat model of CCl_4_-induced cirrhosis. These effects were enhanced by genetically engineering rHGF into transplanted BM-MSCs. Overall, our findings suggest that transplantation of rHGF gene-modified rat-derived BM-MSCs might be a useful and affordable therapeutic option for clinical management of liver cirrhosis.

## Materials and methods

### Liver cirrhosis rat model induced by CCl_4_

We purchased male Sprague-Dawley rats at 1 month of age from the animal facility of the Second Affiliated Hospital of Harbin Medical University. All the rats were maintained in an air-conditioned vivarium with 12/12 h light/dark cycles. The experimental model of liver cirrhosis was induced by intraperitoneal injections of 50% CCl_4_ diluted in olive oil (5.0 ml/kg) for 30 consecutive days, while controls were mock-conditioned by intraperitoneal injections of saline^16,17^. Four CCl_4_-treated experimental and saline-treated control animals were examined after 4 weeks to validate the establishment of the liver injury model. Other CCl_4_-treated animals were randomly divided into four groups that received an intravenous (tail vein injection) infusion of HGF-modified BM-MSCs (1.0 × 10^6^ cells/rat) (HGF-BM-MSC group), unmodified BM-MSCs (1.0 × 10^6^ cells/rat) (BM-MSC group), HGF, or saline (*n* = 5/group). These animals were monitored for 4 weeks post intravenous treatment. Additionally, we confirmed that all experiments and methods were performed in accordance with the relevant ethical guidelines and regulations, and they were approved by the Experimental Center of the Second Affiliated Hospital of Harbin Medical University.

### Preparation of BM-MSCs for cell transplant therapy

The preparation of normal BM-MSCs from donor rats has been described previously^16,17^. Briefly, femoral bone marrow was obtained from 1-month-old rats, washed with PBS, diluted with an equal amount of Percoll separating medium and centrifuged at 2500 rpm for 20 min. The cell sediment was harvested, reconstituted with DMEM/F12 medium (DMEM/F12, 10% FBS, 100000 U/L penicillin, pH = 7.4), and cultured in a bottle with a beginning density of 5 × 10^5^ cells/ml. The medium was changed on the third day and refreshed every 2 days until the third passage of cells ready for transduction. Cultured BM-MSCs were transduced (co-culture) with either rHGF (Rat HGF Gene ID: 24446, MOI = 80) or blank adenovirus at a multiplicity of infection of at least 50 for 48 h. The transduction efficiency, which was the ratio of EGFP-positive cells to total cell numbers, was evaluated using a BX51 fluorescence microscope (Olympus, Japan). The transduced cells were sorted by puromycin dihydrochloride (1 μg/ml, Thermo Fisher Scientific), which was added to the culture medium, passaged and screened continuously until the third generation. After screening, the cells were washed three times with saline, collected and frozen for tail vein injections. The rHGF gene expression and blank adenoviral vectors that are provided commercially by the Shanghai JiKai Chemical Technology have the following characteristics: (1) labelled with EGFP, (2) resistant to puromycin dihydrochloride, and (3) express HGF protein. The viral vector used in the study is shown in Fig. 2i.

### Dil-Ac-LDL uptake and bonding of FITC-CEA-1

BM-MSCs at the second passage were seeded in six-well tissue plates with nutrient solution. After 2 days of incubation, according to manufacturer’s protocol, the cells were incubated with 4 μg/ml Dil-Ac-LDL (Molecular Probes, Eugene, OR, USA) and 50 μg/ml *Ulex europaeus* agglutinin (UEA-1, Sigma, St. Louis, MO, USA), and observed using a BX51 fluorescence microscope (Olympus, Japan)^31^.

### Osteogenic and adipogenic differentiation assays

BM-MSCs in the third passage were seeded in culture medium at 3 × 10^4^ cells/cm^2^ in six-well tissue culture plates. After incubation for 24 h, the growth medium was changed to osteogenic and adipogenic differentiation medium, according to the manufacturer’s protocol^32^. Cells were then cultured in a 5% CO_2_ atmosphere at 37 °C, and the osteogenic and adipogenic differentiation medium was replaced every 3 days. Following incubation for 14 days, osteogenic and adipogenic differentiation was verified by Alizarin Red (Sigma-Aldrich) staining and Oil Red O (Sigma-Aldrich) staining.

### Blood and tissue preparation

Rats were killed by narcotisation with chloral hydrate (0.4 ml/100 g, i.p.) 28 days after tail vein infusion treatments. Trunk blood was obtained from the vena cava for serological tests, while the liver was removed and divided into two halves that were snap-frozen for biochemical and immerse-fixed in 4% buffered formalin for histological studies. Additionally, during the 1-month experimental period, tail vein blood was drawn at day 0 and the end of the first (day 7), second (day 14), third (day 21) and fourth (day 28) weeks after intravenous treatments. The samples were used to measure hepatic enzymes in serum.

### Western blot

Frozen liver samples were homogenised with tissue extraction buffer and EGFP-positive BM-MSCs containing protease inhibitors (Beyotime, China), and the resulting lysates were centrifuged at 12,000 g at 4 °C for 10 min. The supernatants were collected, and the protein concentration was measured. Extracts containing 20 μg proteins were loaded onto 5% SDS-PAGE gels followed by electrophoresis. We transferred the separated proteins to nitrocellulose membranes and then incubated the membranes with primary antibodies to β-actin (1:1000), ALB (ab207327, Abcam, UK, 1:200), CK18 (ab133263, Abcam, UK, 1:200), HNF-4α (ab41898, Abcam, UK, 1:200) and HGF (ab83760, Abcam, UK, 1:200) overnight at 4 °C. the membranes were further incubated with horseradish peroxidase (HRP)-conjugated secondary antibodies (1:20,000; Bio-Rad Laboratories) and treated with the ECL Plus Western Blotting Detection Kit. Finally, we captured Immunoblot images using the Omega-Lum G imaging system.

### HE and picrosirius red staining

Fixed liver samples were paraffin-embedded and prepared into 10 sets of 5-μm-thick sections with a consistent distance of ~1 mm. Sections were stained with HE following dewaxing to assess the degree of hepatic fibrosis. For collagen evaluation, sections were stained with picrosirius red in strict accordance with the manufacturer’s instructions^33^. Images of tissue sections were captured with a BX51 microscope (Olympus, Tokyo, Japan) and were semi-quantitatively determined by Image-Pro Plus (Media Cybernetics, USA).

### Immunofluorescence and immunohistochemical analyses

Immunohistochemical analysis of the liver sections was conducted as described previously^34^. The images were captured by a Nikon microscope (Japan) under a high magnification (×20), and positively labelled cells were semi-quantitatively analysed by Image-Pro Plus (Media Cybernetics, USA). Other sets of sections were dewaxed and subjected for immunofluorescence labelling of ALB (Santa Cruz; 1:100), CK18 (Abcam, 1:200) and HNF-4α (Abcam, 1:100). Sections were incubated with the primary antibodies at the same working dilutions as noted above overnight at 4 °C using normal donkey serum (5%) to decrease nonspecific binding. After rinses with PBS, the sections were incubated with Alexa Fluor® 594-conjugated donkey secondary antibodies (1:200, Invitrogen, USA) for 1 h at room temperature. We subsequently stained the sections with DAPI (4,6-diamidino-2-phenylindole) and used an anti-fading mounting medium before microscopic examination.

### Real-time quantitative PCR

Total RNA in frozen liver tissues was extracted by a TRIzol extraction kit (Invitrogen, Carlsbad, CA, USA) following the manufacturer’s protocol. The cDNAs were generated by a commercial kit (Bioneer, Korea) in reverse transcription reactions. The template DNA was amplified in PCR reactions according to the instructions of a commercial kit (Bioneer, Korea) with the following primers: HNF-4α S: 5′-TGGATATGGCCGACTACAGC-3′, A: 5′-ACCTTCAGATGGGGACGTGTCA-3′^35^, CK18 S: 5′-TGGTACTCTCCTCAATCTGCTG-3′, A: 5′-CTCTGGATTGACTGTGGAAGTG-3′^29^, ALB S: 5′-TCAACGTCAGAGCAGAGAAGC-3′, A: 5′-AGACTGCCTTGTGTGGAAGACT-3′^29^. The amplification reaction was performed for 15 min at 95 ℃ and followed by 40 amplification cycles of 5 s at 95 °C and 30 s at 60 °C. The mRNA expression levels of the target genes were normalised to GAPDH expression. The mean relative gene expressions were detected, and differences were calculated using the 2^−ΔΔCT^ method^36^. It is worth noting that the mRNA expression levels of the treatment groups refer to the mRNA expression levels relative to the control group.

### Serological markers of liver functionality and hydroxyproline determination

Blood samples collected at selected time points were divided into 2 groups. Samples from one group were kept at room temperature for 2 h and then at 4 °C overnight, with the sera collected and frozen. Sera from the groups were thawed and centrifuged at 3000 rpm for 10 min prior to testing. Serum levels of AST, ALT and TBIL were then tested with a Roche Modular DPP automatic biochemical analyser (facilitated in the 2nd Affiliated Hospital of Harbin Medical University). Samples in the other group were stored at 4 °C in a refrigerator overnight and then centrifuged at 1000 × *g* for 20 min. Serum showing lysis of the red blood cells was excluded from the study. Total collagen content was detected by measuring the hydroxyproline level in serum samples using a Hydroxyproline Assay Kit (CEA621Ge 96T, Cloud-Clone Corp) following the manufacturer’s instructions.

### Statistical analyses and figure preparation

All the data are displayed as the means ± SDs. Statistical analyses were performed by Wilcoxon rank-sum test and ANOVA of a completely randomised design and a three-factor factorial design. A Bonferroni test was used for multiple comparisons. All statistical analyses were performed using SPSS software (version 24.0, Chicago, IL). The minimal level for a significant difference was set at *p* < 0.05. All figures were prepared using Photoshop 7.1 and GraphPad Prism 5.0.
